# Australasian paediatric gastroenterologist practices of coeliac disease diagnosis before and during the COVID‐19 pandemic

**DOI:** 10.1111/jpc.16227

**Published:** 2022-09-23

**Authors:** Shaun S C Ho, Helen M Evans, Amin J Roberts, Nikhil Thapar, Shoma Dutt, Kunal Thacker, Usha Krishnan, Chee Y Ooi, Jason Yap, Ajay Sharma, Andrew S Day, the PEDiatric Australasian Gastroenterology Research NEtwork (PEDAGREE)

**Affiliations:** ^1^ Department of Paediatrics University of Otago Christchurch Christchurch New Zealand; ^2^ Department of Paediatric Gastroenterology and Hepatology Starship Child Health Auckland New Zealand; ^3^ Department of Paediatrics: Child and Youth Health University of Auckland Auckland New Zealand; ^4^ Department of Gastroenterology, Hepatology and Liver Transplantation Queensland Children's Hospital Brisbane Queensland Australia; ^5^ School of Medicine University of Queensland Brisbane Queensland Australia; ^6^ Woolworths Centre for Child Nutrition Research Queensland University of Technology Brisbane Queensland Australia; ^7^ Department of Gastroenterology The Children's Hospital at Westmead Sydney New South Wales Australia; ^8^ CHW Clinical School, Children's Hospital Westmead, Faculty of Medicine and Health The University of Sydney Sydney New South Wales Australia; ^9^ Department of Paediatric Gastroenterology Sydney Children's Hospital Randwick Sydney New South Wales Australia; ^10^ School of Clinical Medicine, Randwick Clinical Campus, Discipline of Paediatrics & Child Health University of New South Wales Medicine & Health Sydney New South Wales Australia; ^11^ Department of Gastroenterology and Clinical Nutrition The Royal Children's Hospital Melbourne Victoria Australia; ^12^ Medicine, Dentistry and Health Sciences University of Melbourne Melbourne Victoria Australia; ^13^ Department of Paediatrics, and Paediatric Gastroenterology Joondalup Health Campus, SJOG Midland and Perth Paediatrics Perth Western Australia Australia

**Keywords:** children, ESPGHAN, non‐biopsy

## Abstract

**Aim:**

To explore the perceptions and practices of Australasian paediatric gastroenterologists in diagnosing coeliac disease (CD) before and during the COVID‐19 pandemic.

**Methods:**

Paediatric gastroenterologists in Australasia were invited via email to complete an anonymous online questionnaire over a 2‐week period in 2021.

**Results:**

The questionnaire was completed by 39 respondents: 33 from Australia and six from New Zealand (NZ) equating to a 66% response rate. Thirty‐four (87%) of the 39 respondents reported they currently practised non‐biopsy diagnosis of CD in eligible children, while the rest diagnosed CD using biopsy confirmation only. All NZ respondents practised non‐biopsy CD diagnosis. A majority of responders (76%) who practised non‐biopsy CD diagnosis followed the 2020 European Society for Paediatric Gastroenterology, Hepatology and Nutrition (ESPGHAN) guidelines. Twenty‐two (56%) respondents reported that they started using a non‐biopsy CD diagnosis protocol before the pandemic and did not change their practice during the pandemic, 10 (26%) started diagnosing non‐biopsy CD during the pandemic, 5 (13%) stated their practices of CD were not impacted by the pandemic and 2 (5%) did not respond on whether the pandemic changed their practice.

**Conclusion:**

The majority of Australasian gastroenterologist respondents reported they routinely utilised the 2020 ESPGHAN diagnostic criteria in eligible children; half of them started prior to the pandemic and another quarter started this approach due to the pandemic. A minority of practitioners routinely rely only on biopsy confirmation to diagnose CD.

## What is already known on this topic

1. The European Society of Paediatric Gastroenterology, Hepatology and Nutrition (ESPGHAN) introduced a non‐biopsy coeliac disease (CD) diagnosis pathway for selected children a decade ago and the guidelines were updated in 2020.
2. A range of coeliac serology tests are frequently ordered in children suspected of having CD.

## What this paper adds

1. The majority of Australasian gastroenterologist respondents reported they routinely utilised the 2020 ESPGHAN diagnostic criteria in eligible children to diagnose CD.
2. Half of the Australasian gastroenterologist respondents started practising non‐biopsy CD diagnosis prior to the COVID‐19 pandemic while an additional quarter of clinicians have been practising non‐biopsy CD diagnosis subsequently.
3. A wide variation of practices by Australasian paediatric gastroenterologists in ordering initial screening blood tests for children suspected of having CD, although TTG‐IgA was the most frequently ordered test.

Coeliac disease (CD) is an immune‐mediated enteropathy affecting genetically susceptible individuals in response to recurrent ingestion of gluten‐containing food. The prevalence of CD in Australia and New Zealand (NZ) is estimated at approximately 1.2%^1^, ^2^ with a rising rate among children.^3^

Initial coeliac serology screening is recommended for any individuals suspected of having CD.^4^, ^5^, ^6^ Upon determination of positive serology, a diagnosis of CD can be confirmed by either intestinal biopsy or based upon coeliac serology results alone (non‐biopsy approach). The European Society of Paediatric Gastroenterology, Hepatology and Nutrition (ESPGHAN) introduced a non‐biopsy CD diagnosis pathway for selected children a decade ago^7^ and the guidelines were updated in 2020.^5^ The most recent guidelines maintain its recommendation of non‐biopsy CD diagnosis to include anti‐tissue transglutaminase IgA antibody (TTG‐IgA) 10× upper limit of normal (ULN) and a positive endomysial IgA antibody (EMA) in a second serum sample. However, this iteration no longer requires a need for coeliac HLA typing confirmation or the presence of symptoms suggestive of CD.^5^ Intestinal biopsy is still required in any child who does not fulfil the non‐biopsy criteria.^5^

An earlier survey assessed the perspectives and practices of Australian and NZ paediatric gastroenterologists regarding the diagnosis and management of CD.^8^ That study was conducted towards the end of 2019 prior to the full publication of the 2020 ESPGHAN guidelines.^5^ About 54% of 28 (*n* = 15) respondents solely relied on biopsy confirmation for CD diagnosis, 25% (*n* = 7) offered either biopsy or non‐biopsy confirmation according to parents' wishes and the other 21% (*n* = 6) routinely practised non‐biopsy CD diagnosis.^8^

During the coronavirus disease (COVID)‐19 pandemic, elective medical procedures including endoscopy were variably affected across Australia and NZ. This study hypothesised that the COVID‐19 pandemic had changed the practices of paediatric CD diagnosis by Australasian paediatric gastroenterologists. A cross‐sectional survey was conducted to evaluate the practices of Australasian paediatric gastroenterologists in diagnosing CD before and during the COVID‐19 pandemic. The clinicians' perspectives on initial coeliac screening tests and reasons for uptake or reluctance to practise non‐biopsy CD diagnosis were also sought.

## Methods

### Participants

An email invitation was sent to all practising paediatric gastroenterologists in Australia and NZ to participate in a single anonymous online survey via the Australasian Society of Paediatric Gastroenterology, Hepatology and Nutrition (AuSPGHAN) and Paediatric Network, GESA (Gastroenterology Society of Australia) bulletin board. The AuSPGHAN and Paediatric Network, GESA are closed membership groups. The bulletin board consists of paediatric gastroenterologists and trainees who are currently working or have worked in Australia and NZ. Gastroenterologists who were not working in Australasia at the time of the survey period and trainees were excluded.

The subcommittee of the University of Otago Human Ethics Committee approved the study (Reference number: D21/352).

### Survey

The authors (Executive members of the PEDiatric Australasian Gastroenterology Research NEtwork: PEDAGREE) developed the survey questionnaire (Data S1) and the final version was posted to an online platform (Qualtrics Version 2021: Provo, UT, USA). The survey was open for 2 weeks from 22 November 2021. This period coincided with various COVID‐19 restrictions occurring in Australia and NZ, including reduced endoscopy lists. During the survey period, two reminder emails were sent to encourage participation. The survey collected basic demographics, the impact of endoscopy restrictions at the time of the survey, preferences for coeliac screening blood tests and CD diagnostic methods. Reasons for participants wanting or reluctant to practise non‐biopsy CD diagnosis were explored. Survey responses were excluded if <50% of the questionnaire was completed.

### Statistical analysis

Data were exported from Qualtrics into IBM SPSS Statistics version 28.0 (IBM Corp., Armonk, NY, USA) for descriptive statistical analysis.

## Results

### Respondent background

A total of 42 responses were received and three responders were excluded due to incomplete questionnaires. Thirty‐nine responses (100% completion rate) were included in the final analysis: 33 (85%) from Australia and 6 (15%) from NZ. Based on the known practising paediatric gastroenterologists in Australasia, this provides a 66% response rate (Table 1).

**Table 1:** Background of 39 consultant respondents who completed the survey

	*N* (%)
Australia	33 (85)
New South Wales	12 (36)
Queensland	5 (15)
South Australia	3 (9)
Victoria	8 (24)
Western Australia	5 (15)
New Zealand	6 (15)
Auckland region	5 (83)
Canterbury region	1 (17)
Gastroenterology experience – years after becoming fellow of the RACP college	
≤5 years	8 (21)
6–10 years	9 (23)
11–20 years	13 (33)
20 years	9 (23)
Practice type	
Public hospitals only	9 (23)
Private practice only	7 (18)
Public and private practices	23 (59)
Endoscopy restrictions due to the COVID‐19 pandemic	
Public hospital (total *n* = 32)	
None	16 (50)
<25%	1 (3)
25–50%	2 (6)
51–75%	8 (25)
>75%	5 (16)
Public hospital (total *n* = 32)	
None	22 (73)
<25%	2 (7)
25–50%	3 (10)
51–75%	2 (7)
>75%	1 (3)

RACP, Royal Australasian College of Physician.

Of the 39 respondents, 9 and 7 practised solely in public hospitals and private practice, respectively; while the rest had combined public and private practices. Overall, physicians reported variable restrictions to endoscopy access due to the COVID‐19 pandemic in both countries, with those practising in the public hospital setting reporting more restrictions than those in private practice (Table 1).

### Practices of initial coeliac screening

**Fig. 1:**
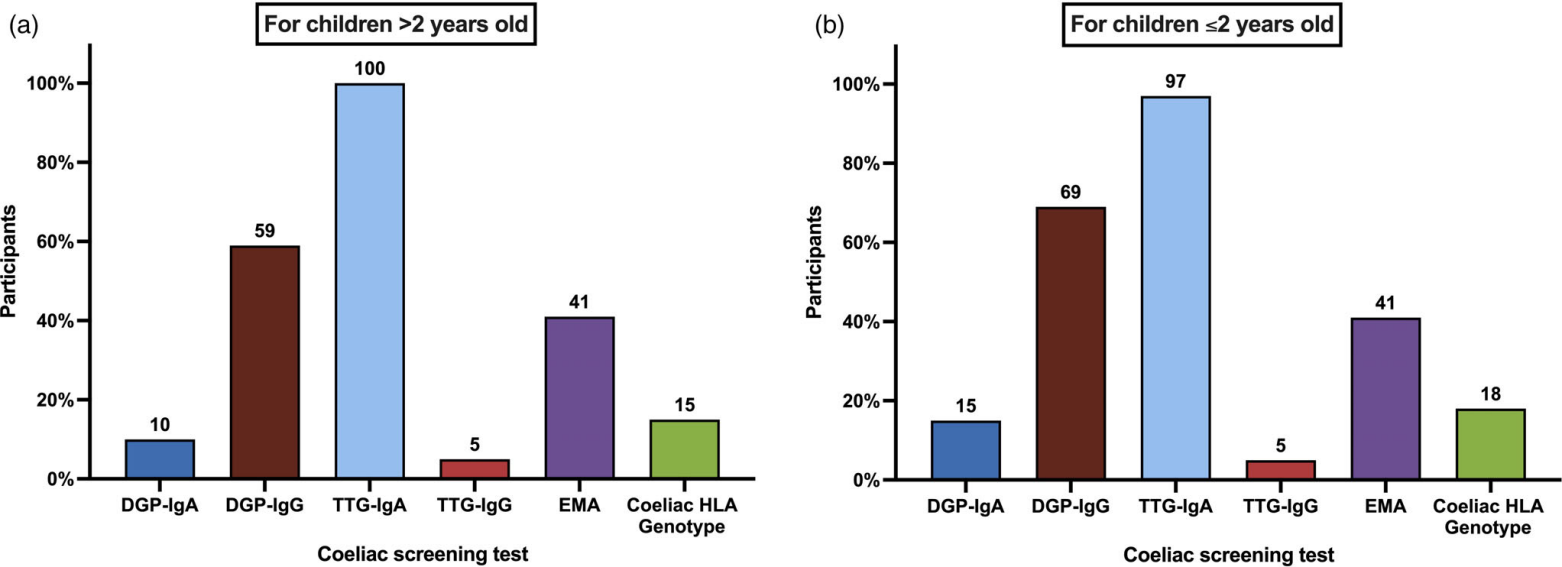
Preferences of 39 Australasian paediatric gastroenterologist in ordering initial screening tests in (a) children aged older than 2 years old, and (b) children aged 2 years and younger. DGP‐IgA, deamidated gliadin peptide IgA antibody; DGP‐IgG, deamidated gliadin peptide IgA antibody; EMA, endomysial IgA antibody; HLA, human leucocyte antigen; TTG‐IgA, anti‐tissue transglutaminase IgA antibody; TTG‐IgG, anti‐tissue transglutaminase IgG antibody.

TTG‐IgA was the most frequently ordered initial coeliac screening test for children of any age: 100% reported by respondents for children >2 years of age and 97% in children ≤2 years of age (Fig. 1). This was followed by deamidated gliadin peptide IgG antibody (DGP‐IgG), utilised by 59% of respondents for children >2 years of age and 69% for children ≤2 years of age. EMA was ordered by 41% of clinicians for children of all ages. Thirty‐six respondents (92%) stated that total Immunoglobulin (Ig) A was routinely ordered as part of their initial coeliac screening tests and 3 others did not request this as their local laboratory services performed total IgA routinely.

When physicians were asked whether other blood tests were ordered with their initial coeliac screening tests, full blood examination and ferritin level were the most requested tests (95% of respondents for both) (Data S2). A minority (5%) of respondents would not order any simultaneous tests.

### The practice of CD diagnosis

At the time of the survey, 34 out of 39 (87%) gastroenterologists reported they practised non‐biopsy CD diagnosis in those children who fulfilled the criteria. The remaining five physicians practised biopsy‐proven CD diagnosis only. When stratified by country, 28 (85%) Australian respondents practised non‐biopsy CD diagnosis compared to all of the NZ respondents.

**Fig. 2:**
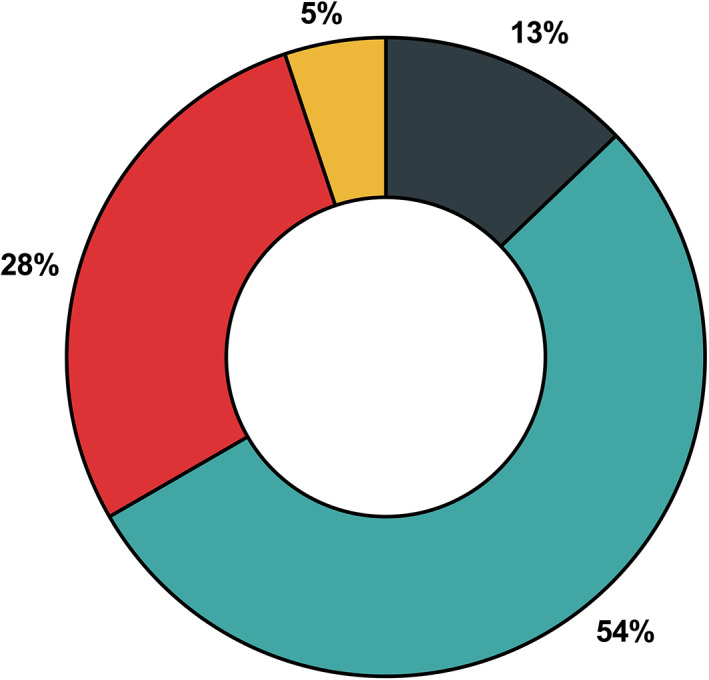
Coeliac disease (CD) diagnostic practices reported by 39 paediatric gastroenterologists before and during the COVID‐19 pandemic. (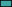) Started practising non‐biopsy CD diagnosis before the pandemic and continued during the pandemic (*n* = 21), (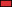) Started practising non‐biopsy CD diagnosis during the COVID‐19 pandemic (One respondent started non‐biopsy CD diagnosis practice during the pandemic because there were sufficient local data to support such practice rather than in response to the pandemic) (*n* = 11), (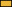) Started practising non‐biopsy CD diagnosis at time of survey but did not report when such practice was started (*n* = 2) and (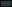) No change in CD practice (biopsy‐proven only) before or during the pandemic (*n* = 5).

When respondents were asked whether the COVID‐19 pandemic had impacted their practice of diagnosing CD in children, half of the respondents (21 out of 39, 54%) stated they started practising non‐biopsy CD diagnosis before the pandemic and did not change their practice during the pandemic (Fig. 2). A quarter of respondents (*n* = 11) reported they started practising non‐biopsy CD diagnosis during the COVID‐19 pandemic. One of the 11 respondents reported that non‐biopsy CD diagnosis practice was started during the pandemic because there was sufficient local data to support such practice rather than in response to the pandemic. Two others (5%) reported they practised non‐biopsy CD diagnosis at the time of the survey but did not report when they started such practice and the rest (*n* = 5, 13%) continued to practise biopsy‐proven CD before and during the pandemic.

**Fig. 3:**
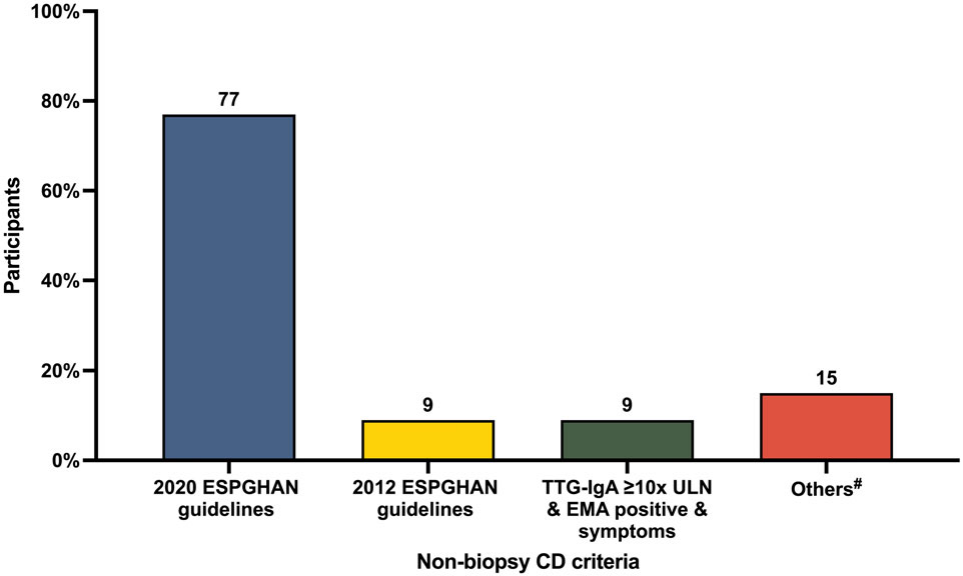
Non‐biopsy coeliac disease (CD) criteria used by 34 physicians who practised non‐biopsy CD diagnosis. Two respondents chose more one criterion in their practice. ^#^Others: TTG‐IgA ≥ 10*x* ULN + symptoms (*n* = 1), TTG‐IgA ≥ 10*x* ULN + EMA positive (one blood sample) (*n* = 1), TTG‐IgA ≥ 10*x* ULN + EMA positive (second blood sample) + coeliac HLA positive (*n* = 1), TTG‐IgA ≥ 10*x* ULN + symptoms + coeliac HLA positive (*n* = 1), TTG‐IgA ≥ 10*x* ULN (2 separate blood samples) + EMA positive (second blood sample) (*n* = 1). CD, coeliac disease; EMA, endomysial IgA antibody; ESPGHAN, European Society for Paediatric Gastroenterology Hepatology and Nutrition; HLA, human leucocyte antigen; TTG‐IgA, anti‐tissue transglutaminase IgA antibody; ULN, upper limit of normal.

Among 34 clinicians who practised non‐biopsy CD diagnosis, 26 (77%) followed the ESGPHAN 2020 guidelines.^5^ Three (9%) continued to follow the ESGPHAN 2012 guidelines,^7^ while another three (9%) followed a variation of the ESPGHAN 2020 guidelines (e.g. using the guidelines only in the setting of consistent symptoms) and the rest reported other variations (Fig. 3). Two respondents selected more than one criterion in their practice of non‐biopsy CD diagnosis. All NZ respondents followed the ESPGHAN 2020 guidelines in diagnosing non‐biopsy CD.

Furthermore, 25 out of 34 (74%) respondents who practised non‐biopsy CD diagnosis would offer biopsy confirmation of CD diagnosis to those patients who fulfilled the non‐biopsy CD criteria. Eight out of 34 (24%) would not offer biopsy confirmation of CD and one respondent did not provide a response.

Among the 31 respondents who requested a second blood sample as part of their criteria in diagnosing CD without a biopsy, 15 (48%) stated they would refer to any laboratory services, another 15 (48%) would refer patients only to specific laboratory services they trust and 1 (4%) would refer patients to the same laboratory service where the serology was initially performed.

Of the four other respondents who accept single blood samples as part of their criteria in diagnosing non‐biopsy CD, two (50%) would refer patients to specific laboratory services they trust and the other two did not provide a response.

In addition, this study explored whether those 34 physicians who utilised non‐biopsy CD diagnosis criteria would specifically exclude certain patients with underlying comorbidities. Most respondents (24 out of 34, 71%) would exclude patients with IgA deficiency, followed by children with type 1 diabetes mellitus (T1DM) and Trisomy 21 (59% and 35% of respondents, respectively) (Data S3). Among the 34 respondents who practised non‐biopsy CD diagnosis, 26 (76%) would proceed to biopsy confirmation in patients with specific comorbidities (as listed in Data S3). Two others would proceed to biopsy confirmation in patients with T1DM who have persistently abnormal coeliac serology without gluten restriction and one other respondent stated that they would be happy to diagnose non‐biopsy CD (based on the ESPGHAN criteria) if they were confident with the coeliac serology assays. The other five respondents reported that they did not apply any exclusion of comorbidity in their non‐biopsy CD diagnosis practices.

### Reasons provided by respondents for wanting or reluctant to practise non‐biopsy CD diagnosis in their practice

The majority of 34 respondents who utilised non‐biopsy CD diagnosis criteria reported that such practice reduced the need for endoscopy (94%) and that it was supported by good evidence (88%) (Data S4). Almost two thirds (62%) of respondents thought that non‐biopsy CD diagnosis would reduce the waiting time for other children needing an endoscopy. Other supportive reasons related to the COVID‐19 pandemic were also reported.

Of the five physician respondents who practised biopsy‐proven CD only, all felt uncertain of the reliability of their local laboratory assays (Data S4). Almost two‐thirds of this group reported personal experience of false‐positive results as a reason to not use non‐biopsy protocols. One of these five responders felt there was insufficient evidence worldwide to support such practice.

## Discussion

This survey found a wide variation of practices by Australasian paediatric gastroenterologists in ordering initial screening blood tests for children suspected of having CD, although TTG‐IgA was the most frequently ordered test. A majority (87%) of these Australasian gastroenterologists reported practising non‐biopsy CD diagnosis at the time of the survey and only a minority continued to solely rely on biopsy‐proven CD diagnosis. Three‐quarters of the respondents reported following the latest 2020 ESPGHAN CD guidelines. However, there was a wide range of perspectives on which comorbidities to exclude from the application of non‐biopsy criteria.

The preferred initial coeliac screening test by this group of gastroenterologists was TTG‐IgA for all children, which is aligned with other guidelines^4^, ^5^, ^6^, ^9^, ^10^, ^11^, ^12^, ^13^ and similar findings to an earlier survey in the Australasian region.^8^ However, other coeliac serologies were commonly ordered concurrently with TTG‐IgA, in particular DGP‐IgG and EMA. The current survey did not explore the rationale for clinicians to order one or other combination of tests.

Interestingly, within the 2 years between the earlier (2019)^8^ and the current study (end of 2021), the percentage of practitioners utilising non‐biopsy CD diagnostic criteria has increased from 21 to 87%. The change in practice was predominantly in the Australian practitioners, whereas the NZ respondents continued to practise non‐biopsy CD diagnosis in both surveys.^8^ It was not possible to track the responses of individual respondents due to the anonymity of both surveys. Notably, there were more respondents who completed the current survey than previously. Nevertheless, it is possible that these physicians may have considered a non‐biopsy diagnosis feasible consequent to the exclusion of coeliac HLA typing and the presence of symptoms suggestive of CD in the updated ESGPHAN guidelines.^5^

In the current study, a quarter of the respondents reported that they started practising non‐biopsy CD diagnosis during the COVID‐19 pandemic. During the current pandemic, the individual states and territories of Australia have regulated their specific public health directions, including endoscopy restrictions in public and private hospitals. Although NZ has had a national public health approach, endoscopy restrictions have varied across regions reflecting local pressures. As the current study reflected the views of practitioners across both countries, it was not surprising that a wide range of endoscopy restrictions was reported. The respondents were asked to comment only on their current level of restrictions (during the 2‐week survey period) as impacts over time would not be able to be recorded clearly. Furthermore, this study did not explore the relationship between the impact of endoscopy restrictions and the respondent's decision to maintain or change practice.

At least three‐quarters of the current surveyed practitioners reported that they used the latest ESPGHAN 2020 guidelines^5^ in their practice of non‐biopsy CD diagnosis, while others reported the use of various diagnostic criteria. The current survey did not explore whether there were local coeliac serology validations performed to support such variation of practice. However, there were different views from the respondents on which laboratory services they would use for a single or second blood sample when it comes to applying the non‐biopsy criteria.

To date, there are currently three known published coeliac serology validations in Australasia that have supported the use of the ESGPHAN non‐biopsy CD criteria in selected children.^14^, ^15^, ^16^ The second blood sample for EMA is recommended in the latest ESPGHAN guidelines to reduce false‐positive cases in those patients who had TTG‐IgA > 10× ULN on their first test.^5^

The respondents gave diverse perspectives on which children with specific comorbidities should be excluded from consideration of diagnosis using a non‐biopsy protocol. Selective IgA deficiency was most felt by respondents to be excluded from the application of non‐biopsy CD criteria: this being consistent with the latest ESPGHAN guidelines.^5^ T1DM was the second most commonly reported comorbidity that the group felt should not be diagnosed using the non‐biopsy approach. This is partly contradictory to the 2020 ESPGHAN guidelines, where the guidelines recommended that non‐biopsy CD diagnosis can be made in symptomatic children with T1DM, but with a conditional recommendation if the child is asymptomatic.^5^ The authors of the guidelines acknowledged that studies with large numbers of children with coexisting T1DM and CD were not included in their literature search.^5^ Despite this, the earlier PRoCeDe study^17^ and a study from NZ^14^ evaluating the ESPGHAN criteria, both found two false‐positive cases. One of the cases in each study was a symptomatic child with T1DM. In contrast, a recent study from Western Australia reported no false positive cases in their validation of the 2020 ESPGHAN criteria; however, the details of the patients' coexisting conditions were not provided.^15^

The current study has some limitations. First, the recorded perspectives reflected the opinions of two‐thirds but not all of the practising Australasian gastroenterologists. However, it did include a range of practice locations across the region. Furthermore, the perspectives provided in this study may be biased towards Australian practices, given there were more participating Australian than NZ physicians.

## Conclusion

In conclusion, a majority of this group of Australasian paediatric gastroenterologists reported that they routinely practised non‐biopsy CD diagnosis in eligible children. Half of the respondents started non‐biopsy diagnosis prior to the COVID‐19 pandemic and the pandemic has further influenced an additional quarter of clinicians to start practising non‐biopsy CD diagnosis. Only a minority continued to solely rely on biopsy confirmation. Of those respondents who practised non‐biopsy CD diagnosis, three‐quarters used the 2020 ESPGHAN guidelines. Given that more Australasian physicians are now utilising non‐biopsy CD diagnostic criteria, a consistent diagnostic approach and standardisation of coeliac serology assays across Australasia will be increasingly important.

## Supporting information


**Data S1.** Questionnaire used for the survey.

**Data S2.** Thirty‐nine respondents' views regarding concurrent tests they would order in addition to the initial screening test in children suspected of having coeliac disease.

**Data S3.** Perspectives of 34 physicians who practised non‐biopsy coeliac disease (CD) on whether non‐biopsy CD criteria should or should not be applied in certain comorbidities.

**Data S4.** Reasons provided by respondents for wanting or reluctant to practise non‐biopsy coeliac disease (CD) diagnosis. (a) Thirty‐four clinicians who routinely practised non‐biopsy CD provided their reasons for wanting such practice. (b) Five respondents who did not practice non‐biopsy CD provided their reasons for not wanting such practice.
