# Supplementary material for: Australasian paediatric gastroenterologist practices of coeliac disease diagnosis before and during the COVID‐19 pandemic
Source: J Paediatr Child Health. 2022 Sep 23;58(12):2280–5. doi: 10.1111/jpc.16227 (PMC10086844; doi:10.1111/jpc.16227)
Supplement: Supplementary file 1 — Data S1. Questionnaire used for the survey. [file JPC-58-2280-s003.pdf]

**Online questionnaire: 'Paediatric gastroenterologist perspectives and practices of coeliac disease diagnosis in Australasia pre and during COVID-19 pandemic'**

**Demographics and practice model:**

1. Are you currently practicing or training in Paediatric Gastroenterology in Australia or New Zealand?
   ☐ Yes
   ☐ No

   1.1. If yes: Where are you currently practicing or training?
   Country:
   ☐ Australia
   - ☐ ACT
   - ☐ NSW
   - ☐ NT
   - ☐ QLD
   - ☐ SA
   - ☐ TAS
   - ☐ VIC
   - ☐ WA

   ☐ New Zealand
   - ☐ Auckland
   - ☐ Canterbury

   1.2. If no: You are not eligible for this survey. We thank you for your interest.

2. How many years since you obtained your fellowship?
   ☐ ≤ 5 years
   ☐ 6 – 10 years
   ☐ 11 – 20 years
   ☐ > 20 years

3. Where do you perform endoscopy? (Select all that apply)
   ☐ Public hospital ☐ Private hospital

   3.1. If public hospital:
   In the **last 2 weeks** of your public hospital practice: Did COVID-related restrictions lead to a reduction or cessation of gastroscopy for confirmation of coeliac disease?
   ☐ Yes ☐ No

   3.1.1. If yes: Please provide an estimate of the extent reduction of your public gastroscopy activity for confirmation of coeliac disease?
   ☐ <25%

☐ 25% - 50%
☐ 51% - 75%
☐ >75%
☐ Others: _____________

3.2. If private hospital:

In the **last 2 weeks** of your private hospital practice: Did COVID-related restrictions lead to a reduction or cessation of gastroscopy for confirmation of coeliac disease?

☐ Yes ☐ No

3.2.1. If yes: Please provide an estimate of the extent reduction of your private gastroscopy activity for confirmation of coeliac disease?

☐ <25%
☐ 25% - 50%
☐ 51% - 75%
☐ >75%
☐ Others: _____________

**Initial screening practice for coeliac disease:**

1. Which of the following coeliac test(s) do you routinely order for children **over** 2 years old suspected of having coeliac disease? (Select all that apply)
   ☐ Deamidated gliadin peptide IgA antibody (DGP IgA Ab)
   ☐ Deamidated gliadin peptide IgG antibody (DGP IgG Ab)
   ☐ Endomysial IgA antibody (EMA)
   ☐ Tissue transglutaminase IgA antibody (TTG IgA Ab)
   ☐ Tissue transglutaminase IgG antibody (TTG IgG Ab)
   ☐ Coeliac HLA genotype
   ☐ Others: ______________

2. Which of the following coeliac test(s) do you routinely order for children **2 years old and under** suspected of having coeliac disease? (Select all that apply)
   ☐ Deamidated gliadin peptide IgA antibody (DGP IgA Ab)
   ☐ Deamidated gliadin peptide IgG antibody (DGP IgG Ab)
   ☐ Endomysial IgA antibody (EMA)
   ☐ Tissue transglutaminase IgA antibody (TTG IgA Ab)
   ☐ Tissue transglutaminase IgG antibody (TTG IgG Ab)
   ☐ Coeliac HLA genotype
   ☐ Others: ______________

3. Do you routinely request a total IgA level if IgA status is unknown?
   ☐ Yes ☐ No

   3.1. If no, does your lab routinely screen for IgA levels whenever coeliac serology is requested?
   ☐ Yes ☐ No ☐ Not necessary

4. Which of the following test(s) do you routinely order in patients suspected of having coeliac disease? (Select all that apply)
   ☐ Full blood count / Full blood examination
   ☐ Electrolytes, urea and creatinine
   ☐ Calcium / phosphate / magnesium level
   ☐ Liver biochemistry
   ☐ Iron studies / Ferritin
   ☐ Vitamin B12
   ☐ Red cell folate
   ☐ Zinc level
   ☐ Vitamin D
   ☐ Thyroid function test / TSH
   ☐ Random blood glucose
   ☐ Hepatitis B serology
   ☐ None
   ☐ Others: __________________

**<u>Coeliac disease diagnostic practice:</u>**

1. In your **current** practice, do you diagnose coeliac disease <u>without</u> biopsy confirmation?
   ☐ Yes ☐ No

   1.1. If yes: Which of the following patient condition(s) (high-risk group) you would **<u>exclude</u>** from using the non-biopsy coeliac disease criteria (even when they fulfilled your criteria)? (Select all that apply)
   ☐ Type 1 diabetes
   ☐ Cystic fibrosis
   ☐ Immunoglobulin A deficiency
   ☐ First-degree family member with coeliac disease
   ☐ Asymptomatic
   ☐ Down syndrome
   ☐ Turner Syndrome
   ☐ Dermatitis herpetiformis
   ☐ None of the mentioned above conditions
   ☐ Others: _________________

   1.1.1. If select any except 'none': What is your approach to these cases?
   ☐ I proceed to intestinal biopsy for confirmation of diagnosis in all mentioned conditions
   ☐ I have different protocol(s) for different condition(s). Please describe: _________________

   1.2. If yes: **Currently**, which of the following criteria would you use to diagnose <u>non-biopsy coeliac disease</u>? (This is in the context of patients not in a high-risk group, i.e. <u>do not</u> have type 1 diabetes, IgA deficiency or cases as identified by you in the earlier question) (Select all that apply)
   ☐ TTG IgA Ab ≥10x ULN **only**
   ☐ TTG IgA Ab ≥10x ULN **AND** symptoms
   ☐ TTG IgA Ab ≥10x ULN **AND** EMA positive (one blood sample)
   ☐ TTG IgA Ab ≥10x ULN **AND** EMA positive (one blood sample) **AND** symptoms
   ☐ TTG IgA Ab ≥10x ULN (first blood sample) **AND** EMA positive (second blood sample) [ESPGHAN 2020 criteria]
   ☐ TTG IgA Ab ≥10x ULN (first blood sample) **AND** EMA positive (second blood sample) **AND** symptoms
   ☐ TTG IgA Ab ≥10x ULN (first blood sample) **AND** EMA positive (second blood sample) **AND** coeliac HLA genotype positive
   ☐ TTG IgA Ab ≥10x ULN (first blood sample) **AND** EMA positive (second blood sample) **AND** coeliac HLA genotype positive **AND** symptoms [ESPGHAN 2012 criteria]
   ☐ Others: _________________

1.2.1. If select any ‘second blood sample’ in Q1.2: Which lab do you send your patient to for the **second** coeliac serology test as part of your <u>non-biopsy coeliac disease</u> diagnosis criteria?

- ☐ To any lab
- ☐ To the same lab where the first coeliac serology was performed
- ☐ To specific lab(s) that I trust. Please provide the lab company name (s): _______
- ☐ Others: _________________

1.2.2. If <u>do not</u> select ‘second blood sample’ in Q1.2: When your patient’s coeliac serology results fulfilled your non-biopsy coeliac disease criteria, which of the following results do you base your diagnosis decision on?

- ☐ Results from <u>any</u> laboratory
- ☐ Results from specific lab(s) that I trust. Please provide the lab company name (s): _______
- ☐ Others: _________________

1.3. If yes: Do you discuss your non-biopsy coeliac disease diagnosis criteria, including advantages and disadvantages with your patients?

- ☐ Yes – all patients regardless of risk(s)
- ☐ Yes – only to patients high-risk group, i.e. patients with type 1 diabetes, cystic fibrosis, etc as mentioned in the earlier question
- ☐ Yes – only to patients who I think are likely to fulfil the non-biopsy criteria
- ☐ Not at all
- ☐ Others: _________________

1.4. If yes: Do you offer biopsy confirmation to those patients who fulfilled the non-biopsy coeliac disease criteria?

☐ Yes ☐ No

1.5. If yes: What is the reason(s) for wanting to use a non-biopsy coeliac disease diagnosis protocol? (Select all that apply)

☐ Validated local serology (prospectively or retrospectively) against the non-biopsy criteria
☐ Good evidence to support such practice
☐ Reduce the need for endoscopy in children
☐ Reduce endoscopy waiting time
☐ Support from peers
☐ Support from hospital
☐ Support from lab services
☐ Reliable results from lab services
☐ COVID pandemic
☐ Parental concern for coming into hospital for endoscopy due to the COVID pandemic
☐ Others: _________________

1.6. If no: What is the reason(s) for not wanting to diagnose coeliac disease without a biopsy? (Select all that apply)

☐ Insufficient evidence worldwide
☐ Insufficient evidence in the location where I practice
☐ Personal experience of false positive results
☐ Unsure of the lab assay reliability
☐ Lack of support from peers
☐ Lack of support from hospital
☐ Lack of support from lab services
☐ Others: _________________

2. During the COVID-19 pandemic (anytime including lockdowns), did your practice of diagnosing coeliac disease change?

☐ I started diagnosing patients as having <u>probable</u> coeliac disease when there was no access to endoscopy. Started patient on a gluten-free diet with the plan to have a gluten challenge at a later stage.
☐ I started diagnosing non-biopsy coeliac disease in those eligible patients <u>during</u> the COVID-19 pandemic.
☐ I started diagnosing non-biopsy coeliac disease <u>before</u> the COVID-19 pandemic. I <u>did not</u> change my non-biopsy coeliac disease criteria during the COVID-19 pandemic for any reason (e.g. limited endoscopy access, etc.).
☐ I started diagnosing non-biopsy coeliac disease <u>before</u> the COVID-19 pandemic. I <u>changed</u> my non-biopsy coeliac disease criteria during the COVID-19 pandemic (e.g. limited endoscopy access, etc.). If yes: what were your criteria **before** the COVID-19 pandemic? ____________
☐ I did not change my practice <u>before or during</u> the COVID-19 pandemic.
☐ Others: _________________

2.1. If select 'I started diagnosing non-biopsy coeliac disease in those eligible patients <u>during</u> the COVID-19 pandemic.' or 'I started diagnosing non-biopsy coeliac disease

before the COVID-19 pandemic. I changed my non-biopsy coeliac disease criteria during the COVID-19 pandemic (e.g. limited endoscopy access, etc.).':
Do you intend to change your diagnostic practice following the COVID-19 pandemic?

☐ Yes ☐ No

2.1.1. If yes:

What would be your approach?

___________________

**Comments:**

1. Please provide any additional comments:

______________________________________________________________________

**END OF QUESTIONS**
