# Supplementary figures and images for: Australasian paediatric gastroenterologist practices of coeliac disease diagnosis before and during the COVID‐19 pandemic
Source: J Paediatr Child Health. 2022 Sep 23;58(12):2280–5. doi: 10.1111/jpc.16227 (PMC10086844; doi:10.1111/jpc.16227)

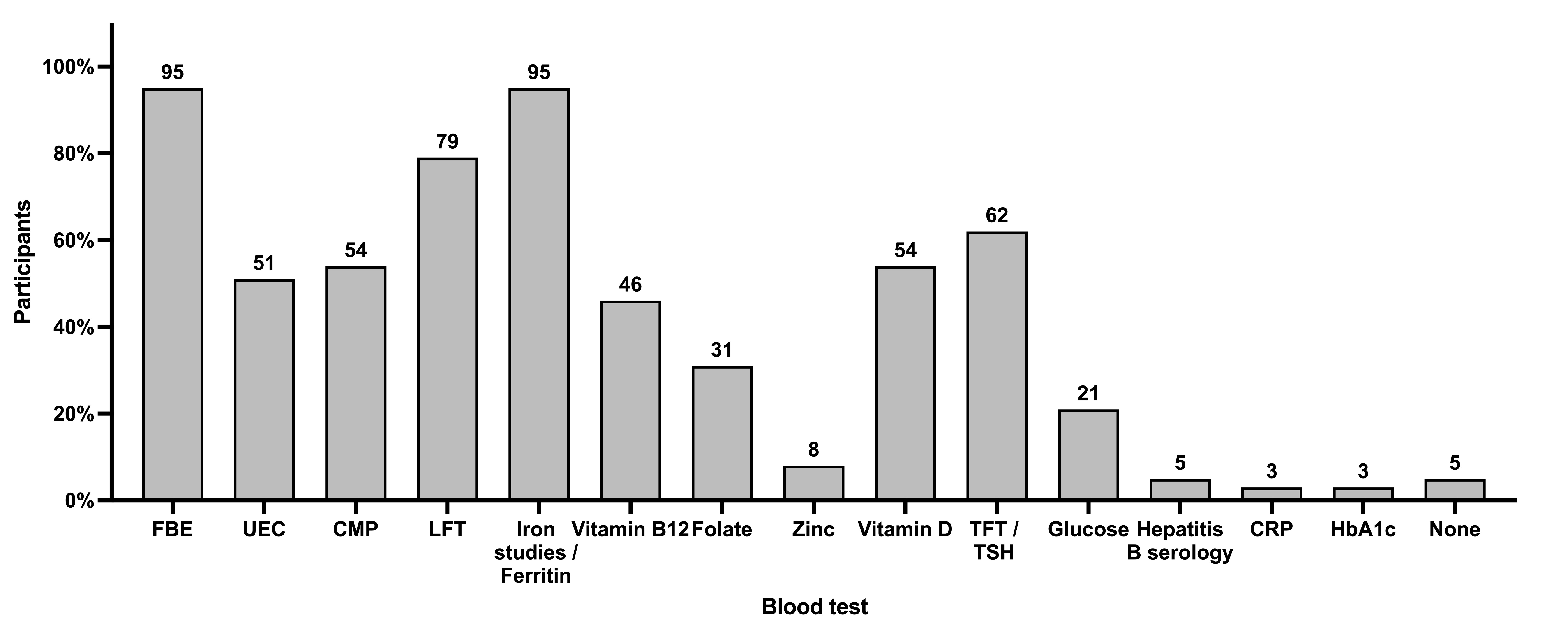

Supplement: Supplementary file 2 — Data S2. Thirty‐nine respondents' views regarding concurrent tests they would order in addition to the initial screening test in children suspected of having coeliac disease. [file JPC-58-2280-s004.jpg]

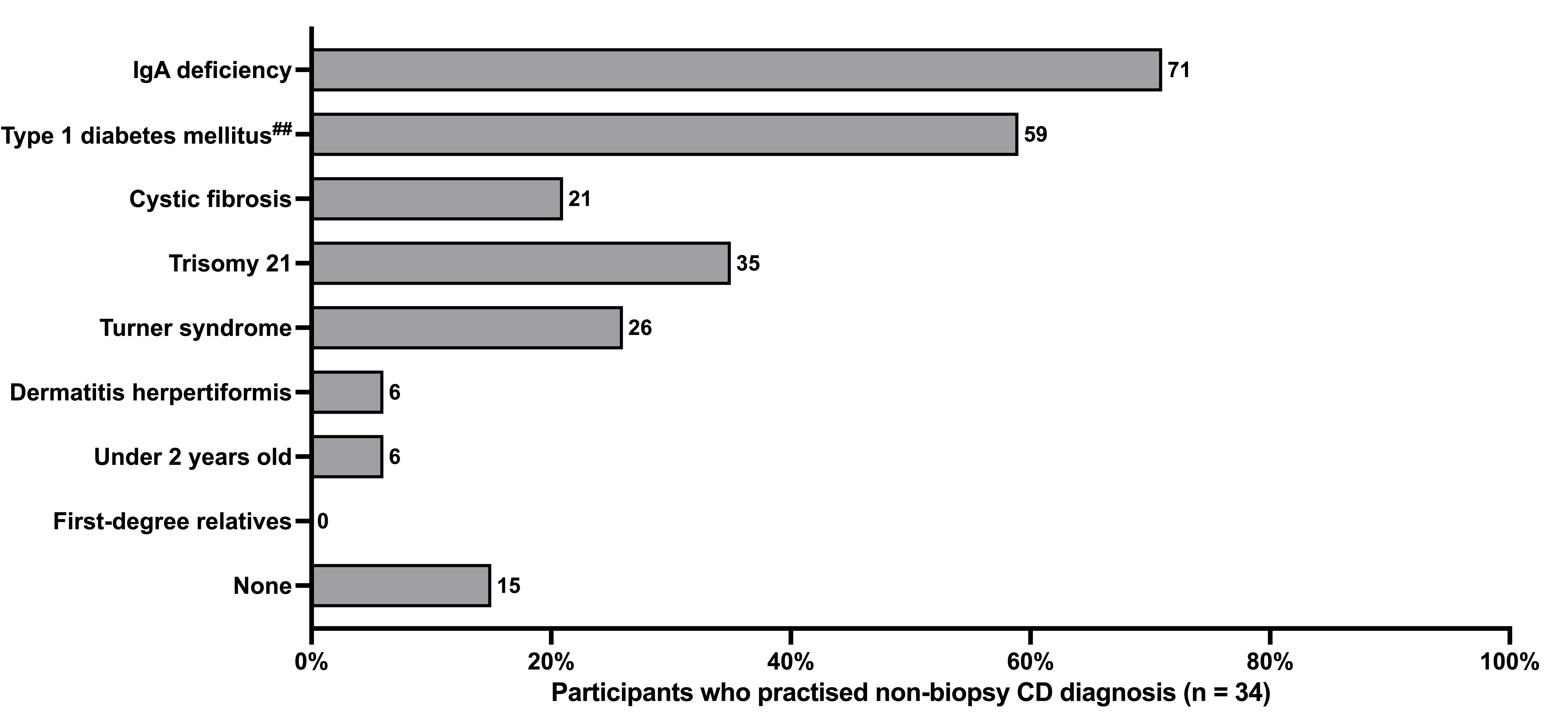

Supplement: Supplementary file 3 — Data S3. Perspectives of 34 physicians who practised non‐biopsy coeliac disease (CD) on whether non‐biopsy CD criteria should or should not be applied in certain comorbidities. [file JPC-58-2280-s002.jpg]

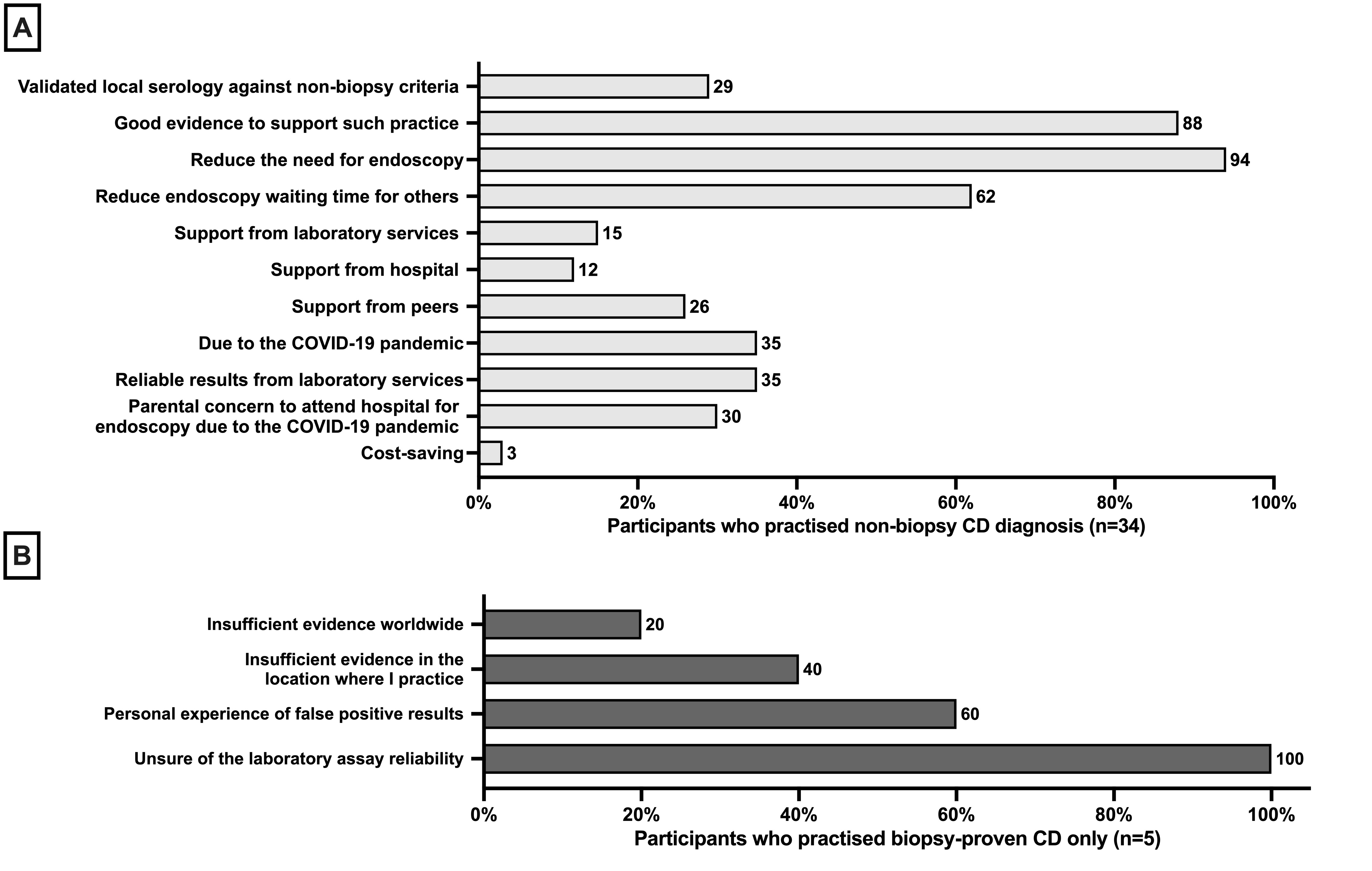

Supplement: Supplementary file 4 — Data S4. Reasons provided by respondents for wanting or reluctant to practise non‐biopsy coeliac disease (CD) diagnosis. (a) Thirty‐four clinicians who routinely practised non‐biopsy CD provided their reasons for wanting such practice. (b) Five respondents who did not practice non‐biopsy CD provided their reasons for not wanting such practice. [file JPC-58-2280-s001.jpg]
